# Fighting and Penalty Minutes Associated With Long-term Mortality Among National Hockey League Players, 1967 to 2022

**DOI:** 10.1001/jamanetworkopen.2023.11308

**Published:** 2023-05-10

**Authors:** Charles A. Popkin, Cole R. Morrissette, Thomas A. Fortney, Kyle L. McCormick, Prakash Gorroochurn, Michael J. Stuart

**Affiliations:** 1 Center for Shoulder, Elbow and Sports Medicine, Columbia University Medical Center, New York, New York; 2 Department of Biostatistics, Columbia University Medical Center, New York, New York; 3 Department of Orthopedic Surgery, Mayo Clinic, Rochester, Minnesota

## Abstract

**Importance:**

National Hockey League (NHL) players are exposed to frequent head trauma. The long-term consequences of repetitive brain injury, especially for players who frequently engage in fighting, remains unknown.

**Objective:**

To investigate the mortality rates and causes of death among NHL enforcers with more career fights and penalty minutes as compared with matched controls.

**Design, Setting, and Participants:**

This matched cohort study examined 6039 NHL players who participated in at least 1 game in the seasons between October 11, 1967, and April 29, 2022, using official NHL data. Cohorts designated as enforcer-fighter (E-F) and enforcer-penalties (E-P) were selected. The E-F cohort consisted of players who participated in 50 or more career fights (n = 331). The E-P cohort included players with 3 or more penalty minutes per game (n = 183). Control-matched NHL players were identified for each E-F player (control-fighter [C-F]) (n = 331) and each E-P player (control-penalties [C-P]) (n = 183).

**Exposures:**

Fighting and penalty minutes were both used as proxies for head trauma exposure. Players with significantly increased exposure to fighting and penalties (E-F and E-P cohorts) were compared with NHL players with less frequent exposure to head trauma (C-F and C-P cohorts).

**Main Outcomes and Measures:**

Mortality rates and age at death of the enforcer and control cohorts, and their causes of death using data obtained from publicly available sources such as online and national news sources, including NHL.com.

**Results:**

Among the 6039 NHL players identified (mean [SD] age, 47.1 [15.2] years), the mean (SD) number of fights was 9.7 (24.5). The mortality rates of E-F and C-F players (13 [3.9%] vs 14 [4.2%], respectively; *P* = .84) or E-P and C-P players (13 [7.1.%] vs 10 [5.5%]; *P* = .34) were not significantly different. The mean (SD) age at death was 10 years younger for E-F players (47.5 [13.8] years) and E-P players (45.2 [10.5] years) compared with C-F players (57.5 [7.1] years) and C-P players (55.2 [8.4] years). There was a difference in causes of death between the control and enforcer players (2 neurodegenerative disorders, 2 drug overdoses, 3 suicides, and 4 vehicular crashes among enforcers vs 1 motor vehicle crash among controls; *P* = .03), with enforcers dying at higher rates of overdose (2 of 21 [9.5%] vs 0 of 24) and suicide (3 of 21 [14.3%] vs 0 of 24) (*P* = .02).

**Conclusions and Relevance:**

The findings of this matched cohort study indicate that there is no difference in overall mortality rates between NHL enforcers and controls. However, being an enforcer was associated with dying approximately 10 years earlier and more frequently of suicide and drug overdose.

## Key Points

**Question:**

Do National Hockey League (NHL) enforcers with more career fights or penalty minutes differ in mortality rates or cause of death when compared with control NHL players?

**Findings:**

This matched cohort study of 6039 NHL players from 1967 to 2022 confirmed that NHL enforcers died at similar rates but approximately 10 years earlier when compared with NHL controls. Furthermore, the causes of death in the 21 enforcers included neurodegenerative disorders, drug overdose, suicide, and motor vehicle crashes, whereas only 1 of the 24 age-matched controls died of any of these causes (motor vehicle crash).

**Meaning:**

These findings suggest that NHL enforcers with 50 or more career fights or 3 or more penalty minutes per game died 10 years earlier and more often of drug overdose and suicide when compared with age-matched NHL player controls.

## Teaser

This matched cohort study examines the mortality rates and causes of death among National Hockey League enforcers compared with matched controls from 1967 to 2022.

## Introduction

Fighting has been implicitly accepted in the National Hockey League (NHL) for more than 100 years.^1,2,3^ Players participating in a fight are reprimanded with a brief 5-minute penalty but return to play shortly after their altercation.^4,5^ Proponents of fighting argue that it promotes ticket sales, deters other acts of violence, and positively impacts team momentum.^5,6,7^ Meanwhile, others have noted the deleterious effects of repetitive head trauma.^3,8,9,10,11^ Enforcers in the NHL are players who engage in fights to intimidate opponents or gain momentum for their team.^12^ The premature deaths of multiple NHL enforcers, including Rick Rypien (aged 27 years), Derek Boogaard (aged 28 years), Wade Belak (aged 35 years), Steve Montador (aged 35 years), and Bob Probert (aged 45 years), have drawn attention to the potential health consequences of fighting.^13^

The risks of fighting and repetitive head trauma are a growing concern among player safety advocates.^10,14,15,16,17^ While fighting accounts for 9% of acute concussions in NHL players,^18^ repetitive head trauma may contribute to the development of headaches, depression, personality changes, and cognitive deficits. Later effects include increased risk of neurodegenerative diseases, including chronic traumatic encephalopathy (CTE) and even suicide.^19,20,21,22^ A recent examination of all-cause mortality comparing National Football League (NFL) players with Major League Baseball players demonstrated that NFL players had an elevated all-cause mortality.^23^ A similar analysis of mortality rates in NHL enforcers is urgently needed to better characterize the long-term association of repetitive head trauma from fighting.

To better understand the association of fighting with NHL player safety, we aimed to examine the mortality rates and causes of death among NHL enforcers compared with matched NHL controls. An NHL enforcer was defined as any player with 50 or more career fights and/or 3 or more penalty minutes per game.

## Methods

This project was conducted at the New York Presbyterian–Columbia University Irving Medical Center (NYP-CUIMC). The study was approved by the NYP-CUIMC’s Institutional Review Board, which did not require informed consent owing to the use of retrospective and publicly available data. This observational cohort study adheres to the guidelines as outlined by the Strengthening the Reporting of Observational Studies in Epidemiology (STROBE) protocol.

### Data Collection

Statistical data for all NHL players who participated in the seasons between October 11, 1967, and April 29, 2022, were collected from the official league statistical resource.^24^ The 1967-1968 season was used as the initial season because it was the NHL expansion year and more accurately reflects present-day NHL conditions. Specific metrics collected for each player included position, date of birth, games played, goals, assists, points, penalty infraction minutes (PIM), PIM per games played (PIM/GP), penalties drawn, penalties taken, net penalties, minor penalties, major penalties, misconduct penalties, game misconduct penalties, birth city, nationality, height, weight, draft year, draft round, overall draft pick, and first season played. Fighting data were compiled from the official NHL box scores. Racial and ethnic data were not incorporated for this study.

All players who participated in the NHL seasons between October 1967 and April 2022 were then filtered to establish the enforcer-fighter (E-F) cohort. The E-F players were defined as those with at least 50 career fights. A total of 331 E-F individuals were identified. Fifty fights were used as the cutoff proxy for repetitive head trauma exposure to capture a cohort of players with the longevity and grit of a seasoned enforcer. Based on the histogram distribution of career fights for all players, it appeared that 50 fights would appropriately eliminate players with inadequate fighting exposure.

The control-fighter (C-F) cohort was then established by matching each individual in the E-F cohort to another NHL player with fewer than 50 career fights but with similar baseline characteristics based on prespecified variables, including date of birth, total number of games played, height, weight, and position played. This matching process was completed via MATLAB computer programming, version R2022a (MathWorks), to establish controls via a best-fit model. Each of the variables to be controlled for was normalized to carry equal weight, and the matching algorithm filtered through all NHL players to find the control individual with the smallest summed difference between control variables. This method was used to reduce possible user bias when selecting controls. A total of 331 C-F individuals were selected.

The same experimental and control selection and matching methods were then repeated, this time focusing on PIM/GP. The experimental cohort was termed enforcer-penalties (E-P) and included all players with a mean of 3 or more PIM/GP. This group of enforcers did not necessarily experience 50 fights in the NHL but were exposed to significant cumulative physicality and a mean of 1 fight nearly every other game. A total of 183 E-P individuals were selected. By using the same matching variables and method as previously outlined, a total of 183 control-penalties (C-P) individuals were selected.

With the 2 enforcer (E-F and E-P) cohorts and the 2 control (C-F and C-P) cohorts selected, the mortality data for each player were then collected manually using a combination of publicly available sources and were cross-referenced for validation. In nearly all instances, local newspaper sources from the player’s state of residence were correlated with other regional online news publications or national news sources, including NHL.com, regarding the death of a player. For players who had died, their age at death and cause of death was recorded (if reported), and the remaining players were marked as alive.

### Statistical Analysis

Comparisons between the enforcer and control cohorts were achieved using *t* tests with 2-tailed distribution and heteroscedastic variance. Unless otherwise specified in the following figures, all *P* values reported were calculated using a 2-sided *t* test comparison. Spearman correlation coefficients were calculated for the variables of interest as well. For causes of death, the Fisher exact test was used. Level of significance was set at α = .05 with analysis via MATLAB, version R2022a (MathWorks). Unless otherwise indicated, data are expressed as mean (SD).

## Results

**Figure 1.**
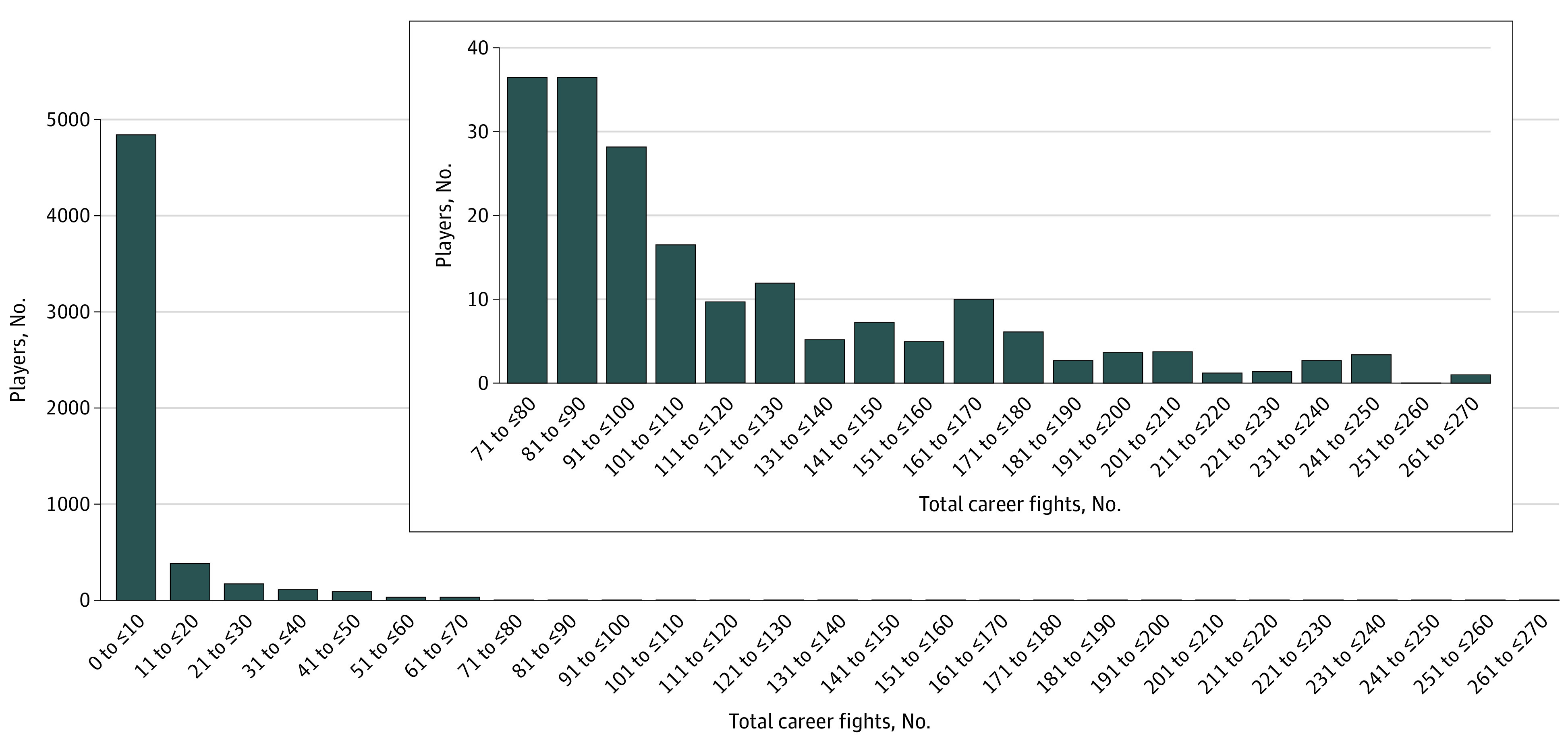
Histogram Analyzing the Distribution of the Total Career Fights for All 6039 National Hockey League Players Between October 1967 and April 2022

A total of 6039 players were identified as having participated in an NHL game between 1967 and 2022. The mean number of career fights for all players was 9.7 (24.5); the mean number of games played was 285.6 (338.9); and the mean age was 47.1 (15.2) years. A total of 331 NHL players were identified as having participated in at least 50 career fights; these 331 players were categorized as the E-F cohort. An equal number of control-matched players with fewer than 50 career fights were also identified and labeled as the C-F cohort. A histogram examining the distribution of the summed career fights for each player can be found in Figure 1.

When the E-F and C-F cohorts were compared, the control-matched variables (including date of birth, total number of games played, height, weight, and position played) were not statistically different between the E-F and C-F players (Table 1). The percentages of E-F and C-F players currently alive also were not statistically different (318 [96.1%] and 317 [95.8%], respectively; *P* = .84). However, when the E-F and C-F players who have died were compared (13 [3.9%] and 14 [4.2%], respectively), E-F players were approximately 10 years younger at death E-F, 47.5 [13.8] years; C-F, 57.5 [7.1] years; *P* = .03). Furthermore, despite having similar overall draft pick positions (Table 1), E-F players had significantly more career penalty minutes (E-F, 1371.0 [645.9] minutes; C-F, 442.1 [290.7] minutes; *P* < .001) with significantly fewer career goals (E-F, 90.0 [112.9] goals; C-F, 134.4 [130.5] goals; *P* < .001) and significantly fewer career assists (E-F, 146.5 [154.8] assists; C-F, 223.1 [187.3] assists; *P* < .001).

**Table 1.** Comparison of Enforcer-Fighter and Control-Fighter for Variables of Interest^a^

Variable	Enforcer-fighter (n = 331)	Control-fighter (n = 331)	*P* value
**Controlled**			
Year born	1969 (10.1)	1969 (10.3)	.99
No. of games played	668.1 (308.6)	661.1 (307.5)	.77
Height, cm	186.9 (5.3)	186.9 (4.8)	.97
Weight, kg	94.6 (7.3)	93.7 (6.8)	.12
Forward, No. (%)	222 (67.1)	222 (67.1)	.91
**Experimental**			
No. of fights^b^	94.2 (44.8)	7.1 (5.9)	<.001
Currently alive, No. (%)	318 (96.1)	317 (95.8)	.84
Age at death, y	47.5 (13.8)	57.5 (7.1)	.03
No. of goals^b^	90.0 (112.9)	134.4 (130.5)	<.001
No. of assists^b^	146.5 (154.8)	223.1 (187.3)	<.001
Penalty infraction minutes^b^	1371.0 (645.9)	442.1 (290.7)	<.001
Penalties drawn	186.6 (72.5)	104.8 (53.6)	.009
Overall draft pick position	67.2 (60.7)	66.8 (69.1)	.94

^a^ Unless otherwise indicated, data are expressed as mean (SD).

^b^ Summed values for each player over their career.

**Figure 2.**
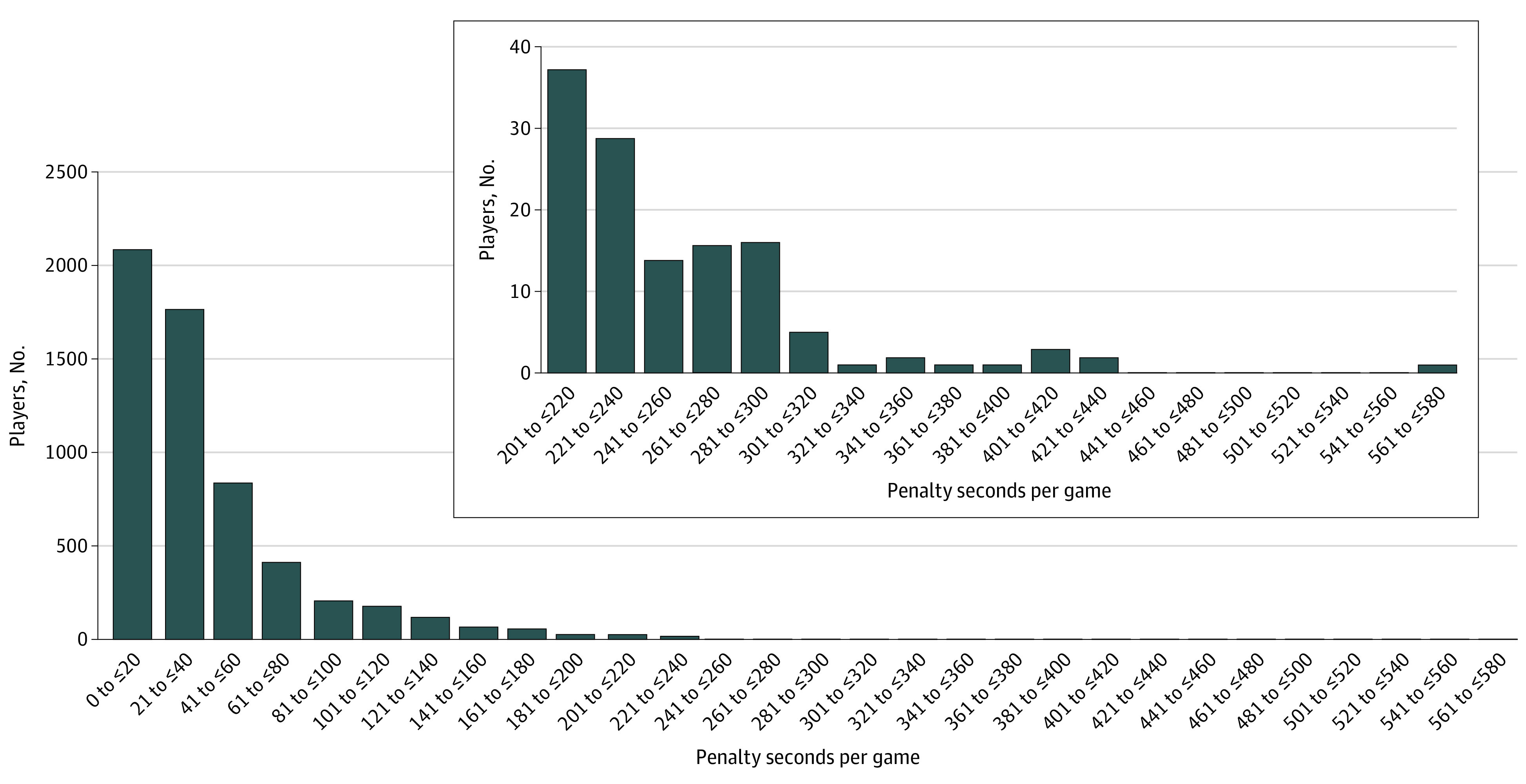
Histogram Analyzing the Distribution of Mean Penalty Seconds per Game for All 6039 National Hockey League Players Between October 1967 and April 2022

A total of 183 NHL players were identified as having a mean of 3 or more PIM/GP; these 183 players were categorized as the E-P cohort. An equal number of control-matched players with no more than 1 PIM/GP were also identified and labeled as the C-P cohort. A histogram examining the distribution of the mean penalty seconds per game for each player can be found in Figure 2.

When the E-P cohort was compared with the C-P cohort, the control-matched variables (including date of birth, total numbers of games played, and position played) were not statistically different between the E-P and C-P players (Table 2). There was a statistically significant but nominally small difference between mean (SD) height (E-P, 186.9 [5.6] cm; C-P, 185.4 [4.8] cm; *P* = .01) and weight (E-P, 93.8 [7.8] kg; C-P, 90.4 [6.3] kg; *P* < .001). The percentage of E-P and C-P players alive at the time of the study was not statistically different (170 [92.9%] and 173 [94.5%], respectively; *P* = .52). However, when the E-P and C-P players who died were compared (13 [7.1%] for E-P vs 10 [5.5%] for C-P), the E-P players were approximately 10 years younger at death (E-P, 45.2 [10.5] years; C-P, 55.2 [8.4] years; *P* = .02). Individuals in the E-P cohort had significantly more career fights than C-P individuals (E-P, 48.4 [64.7] fights; C-P, 0.7 [1.7] fights; *P* < .001). Furthermore, despite having similar overall draft pick positions (Table 2), E-P players had significantly more career PIM (E-P, 643.7 [881.8] minutes; C-P, 50.8 [66.3] minutes; *P* < .001) with significantly fewer career goals (E-P, 15.1 [34.9] goals; C-P, 36.7 [67.2] goals; *P* < .001) and significantly fewer career assists (E-P, 22.7 [46.1] assists; C-P, 56.3 [98.0] assists; *P* < .001). When the number of career fights was analyzed as a continuous variable for the 127 players with more than 1 career fight who died, no statistical significance was achieved (fitted model: age at mortality = 55.04 − 0.071 × [No. of fights]; *P* = .08; *R*^2^ = 0.024) (eFigure in Supplement 1).

**Table 2.** Comparison of Enforcer-Penalties and Control-Penalties for Variables of Interest^a^

Variable	Enforcer-penalties (n = 183)	Control-penalties (n = 183)	*P* value
**Controlled**			
Year born	1969 (10.24)	1970 (11.74)	.25
No. of games played	177.7 (243.9)	205.1 (247.4)	.29
Height, cm	186.9 (5.6)	185.4 (4.8)	.01
Weight, kg	93.8 (7.8)	90.4 (6.3)	<.001
Forward, No. (%)	131 (71.6)	139 (76.0)	.34
**Experimental**			
No. of fights^b^	48.4 (64.7)	0.7 (1.7)	<.001
Currently alive, No. (%)	170 (92.9)	173 (94.5)	.52
Age at death, y	45.2 (10.5)	55.2 (8.4)	.02
No. of goals^b^	15.1 (34.9)	36.7 (67.2)	<.001
No. of assists^b^	22.7 (46.1)	56.3 (98.0)	<.001
Penalty infraction minutes^b^	643.7 (881.8)	50.8 (66.3)	<.001
Overall draft pick position	92.7 (58.9)	88.3 (68.0)	.57

^a^ Unless otherwise indicated, data are expressed as mean (SD).

^b^ Summed values for each player over their career.

**Figure 3.**
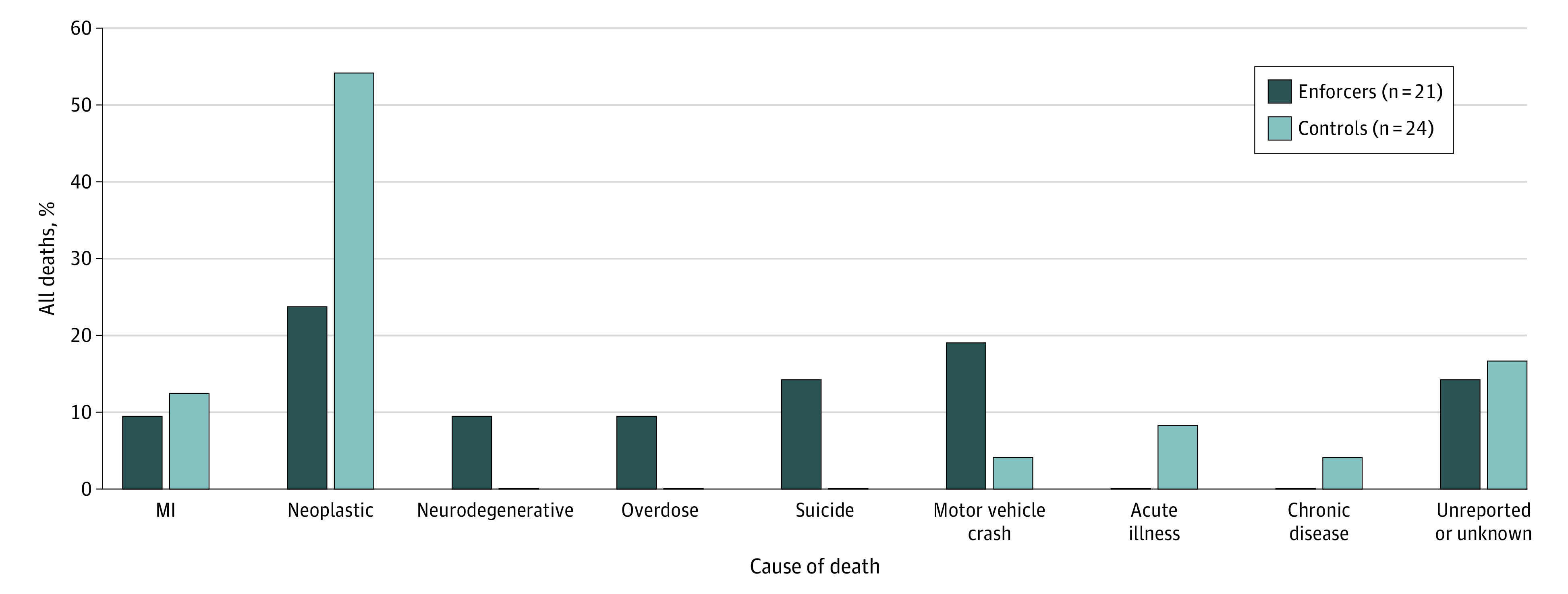
Causes of Death of All Enforcers and Controls Among National Hockey League Players Between October 1967 and April 2022 Enforcers include those who have died from the enforcer-fighter and enforcer-penalties cohorts. Controls include those who have died from the control-fighter and control-penalties cohorts. When the association between player type (enforcer vs control) and cause of death was compared, there was a statistically significant difference between causes of death (21 enforcers vs 24 controls; Fisher exact test, *P* = .03). Furthermore, enforcers died at a significantly higher frequency from overdose and suicide (5 enforcers vs 0 controls; Fisher exact test, *P* = .02). MI indicates myocardial infarction.

The causes of death for enforcers and controls can be found in Figure 3. Enforcers include those who have died from the EF and EP cohorts (includes 21 unique players). Controls include those who have died from the C-F and C-P cohorts (includes 24 unique players). Neoplastic etiology accounted for 13 control deaths (54.2%) and 5 enforcer deaths (23.4%). Furthermore, when the causes of death for enforcers were examined, 2 (9.5%) died of overdoses, 3 (14.3%) died of suicide, and 2 (9.5%) died of neurodegenerative diseases, while no controls died of these causes. Four enforcers (19.0%) died of vehicle-related crashes, whereas only 1 control (4.2%) died of such a crash. When the association between player type (enforcer vs control) and cause of death was compared, there was a statistically significant difference between causes of death (21 enforcers vs 24 controls; Fisher exact test, *P* = .03). Specifically, enforcers died at a significantly higher rate from overdose and suicide (5 enforcers vs 0 controls; Fisher exact test, *P* = .02).

## Discussion

When we examined the all-cause mortality of NHL enforcers playing between 1967 and 2022 (defined by either total career fights or mean PIM/GP), there was no difference in mortality rates between NHL enforcers and NHL controls. However, being an NHL enforcer was associated with an earlier age at death (mean of approximately 10 years earlier) than their controls. Furthermore, when we analyzed the cause of death, we observed that enforcers died more frequently of drug overdose and suicide.

Repetitive subconcussive forces and diagnosed concussions have become a major focus of player safety advocates considering the long-term consequences of these injuries, including CTE.^17,18,21,25^ A concussion monitoring program was established by the NHL and NHL Players Association in 1997 and more recently evolved into a summit where experts analyzed concussion trends, pathophysiology, and management strategies.^26^ Approximately 9% of all documented concussions occur secondary to a fighting incident.^16^ However, the true number of concussions is known to exceed the documented rate, even at the youth level.^27,28,29^ Given that the sole purpose of a fight is to strike an opponent’s head and that NHL players may have additional financial incentives to underreport concussions, it is likely that fighting contributes to a greater number of concussions among NHL players.

In 2010, the NHL demonstrated concern for head trauma and instituted the first penalty for head contact via Rule 48. However, the punishment for head contact was soon reduced by provisions to Rule 48 in 2011 and 2012 (eTable in Supplement 1). This sequence of events was problematic, considering the league was simultaneously acknowledging the dangers of head trauma and reducing the penalty for engaging in dangerous play that resulted in head contact.^30^

Despite the similar mortality rates among NHL enforcers and controls, the younger deaths along with the distinctive causes of death among enforcers are troubling in the context of CTE. Chronic traumatic encephalopathy is confirmed at autopsy by the presence of hyperphosphorylated tau in neurons and has been associated with a clinical syndrome manifested by cognitive decline, behavioral changes, and suicidal ideation.^19,20,21,22,31^ Of the 21 enforcers who died, 11 died of causes often associated with CTE pathology (2 neurodegenerative disorders, 2 drug overdoses, 3 suicides, and 4 motor vehicle crashes). Fighting exposes players to repetitive head trauma and may be associated with increased risk of developing CTE. Therefore, it is not surprising that NHL enforcers died via mechanisms consistent with CTE pathology, including suicide, substance abuse, and motor vehicle crashes.^20,32,33^ Aside from 1 motor vehicle crash, no control group players died of these causes.

It is important to note that the NHL stands alone as the sole professional sports league that does not immediately eject players who fight.^34,35,36^ Major League Baseball, the NFL, and National Basketball Association levy harsh fines and immediate suspensions for any player participating in a fight. While fights currently occur in 1 of every 5.5 NHL games, the frequency has been steadily declining since its peak in 1987 at a mean of more than 1 fight per game.^3,11^ Furthermore, recent studies have refuted the conventional beliefs surrounding the importance of fighting in the NHL. It was recently shown that fighting does not act as a deterrent for lesser infractions,^11,37^ fighting is negatively associated with fan attendance at present,^3^ and fighting does not improve the probability of winning.^9,11^ With declining rates of fighting and a lack of evidence that fighting promotes attendance, winning, or player safety, it is time that the NHL aligns with other professional sports and eliminates fighting.

### Limitations

This cohort study is not without limitations. First, while using all-cause mortality as our primary end point provides a powerful conclusion, it does not demonstrate the true morbidity that fighting may inflict on enforcers. Repetitive head trauma and CTE can result in numerous deleterious effects, including headaches, depression, anxiety, and personality changes, none of which was quantified in our analysis. Second, there are no included data with this study on clinically diagnosed concussions in these ice hockey players. We are using number of fights (≥50) and PIM/GP (≥3) as proxies for repetitive head trauma. Third, while the control-matching method captures many key variables that could differ during an NHL career and can offer an estimate of exposure to head trauma, it is not all inclusive. There are likely variables that could have been included to strengthen our control-matched cohort. Fourth, while the fighting and penalty data were collected for all NHL games, these players had lengthy hockey careers before entering the NHL and likely had important prior exposures from junior hockey. Unfortunately, prior career data from other leagues are not available for all players; therefore, a comprehensive analysis of these players from the beginning of their career is unfeasible. Finally, players who become enforcers may have a different psychological profile at baseline that puts them at risk for substance abuse, suicide, and risky behavior.

## Conclusion

The findings of this matched cohort study suggest that there is no significant difference in overall mortality rates between NHL enforcers (players with ≥50 career fights or ≥3 PIM/GP) compared with control-matched NHL players. However, being an NHL enforcer was associated with dying a mean of 10 years earlier and more frequently of suicide and drug overdose than matched controls. Reemphasis on player safety and improving quality of life after a hockey career should renew discussion to make fighting a game misconduct penalty in the NHL.

## Notes

Supplement 1. **eFigure.** Regression Analysis of Fighting and Mortality **eTable.** Summary of Rule 48 and Its Modification
eReferences

Supplement 2.
Data Sharing Statement
