# Supplementary material for: Fighting and Penalty Minutes Associated With Long-term Mortality Among National Hockey League Players, 1967 to 2022
Source: JAMA Netw Open. 2023 May 10;6(5):e2311308. doi: 10.1001/jamanetworkopen.2023.11308 (PMC10173025; doi:10.1001/jamanetworkopen.2023.11308)
Supplement: Supplement 1. — eFigure. Regression Analysis of Fighting and Mortality eTable. Summary of Rule 48 and Its Modification eReferences [file jamanetwopen-e2311308-s001.pdf]

Supplementary Online Content

Popkin CA, Morrissette CR, Fortney TA, McCormick KL, Gorroochurn P, Stuart MJ. Fighting and penalty minutes associated with long-term mortality among National Hockey League players, 1967 to 2022. *JAMA Netw Open*. 2023;6(5):e2311308. doi:10.1001/jamanetworkopen.2023.11308

eFigure. Regression Analysis of Fighting and Mortality

eTable. Summary of Rule 48 and Its Modification

eReferences

This supplementary material has been provided by the authors to give readers additional information about their work.

© 2023 Popkin CA et al. *JAMA Network Open*.

eFigure. Regression Analysis of Fighting and Mortality

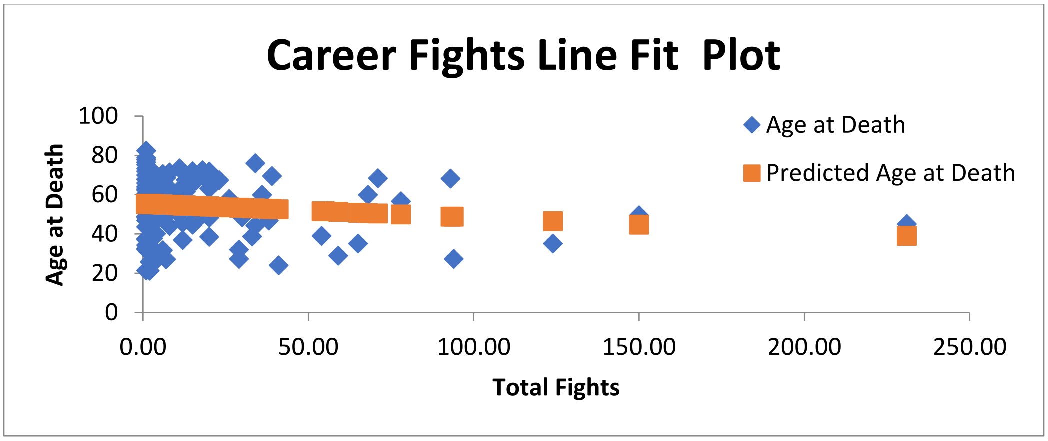

When the number of career fights was analyzed as a continuous variable for the 127 players who had > 1 career fight and died, there was no statistical significance achieved (fitted model: Age at Mortality = 55.04 – 0.071*[fights], p=0.085, R^2 = 0.024). While not statistically significant, this model is not incongruent with our hypothesis of cumulative injury due to fighting. A total of 45.23% of the players in this model had fewer than 5 career fights, and these players are unlikely to have significantly increased risk of mortality from this level of exposure, likely contributing to the lack of significance observed in this regression.

© 2023 Popkin CA et al. *JAMA Network Open*.

eTable. Summary of Rule 48 and Its Modification

In both 2011 and 2012 the repercussions for head contact were reduced, with additional criteria being added in 2012 to reduce the responsibility of the individual initiating the contact.

2010[1]

- Rule 48 Introduced
    - "A lateral or blind side hit to an opponent where the head is targeted and/or the principle point of contact is not permitted"
- **Penalization** – no minor penalty option, mandatory major penalty + game misconduct with optional supplementary penalizations by league/officials

2011[2]

- Rule 48 Modified
    - "A hit resulting in contact with an opponent's head where the head is targeted and the principal point of contact is not permitted."
- **Penalization** – minor penalty, optional major penalty, optional game misconduct, optional supplementary penalizations by league/officials

2012[3] → Present

- Rule 48 Modified
    - Language changed to focus on a "hit resulting in contact with an opponent's head where the head was the main point of contact and such contact to the head was avoidable"
    - Enacted stipulations, all of which mitigate the responsibility of the player initiating the hit. Reduced penalty if the following occur (original language):
        - "The head was not 'picked' as the target to be hit"
        - "The player being hit put themselves in a 'vulnerable' position"
        - "The player being hit changed positions before or during the hit that contributed to head contact"
- **Penalization** – minor penalty, optional major penalty, optional game misconduct, optional supplementary penalizations by league/officials

eReferences

1. National Hockey League Official Rules 2010-2011. Accessed https://www.yumpu.com/en/document/view/3861833/2010-11-nhl-rulebook-national-hockey-league-officials-/3
2. National Hockey League Official Rules 2011-2012. Triumph Books, Chicago, Illinois.
3. National Hockey League Official Rules 2012-2013. Triumph Books, Chicago, Illinois.

© 2023 Popkin CA et al. *JAMA Network Open*.
