# Supplementary material for: Fighting and Penalty Minutes Associated With Long-term Mortality Among National Hockey League Players, 1967 to 2022
Source: JAMA Netw Open. 2023 May 10;6(5):e2311308. doi: 10.1001/jamanetworkopen.2023.11308 (PMC10173025; doi:10.1001/jamanetworkopen.2023.11308)
Supplement: Supplement 2. — Data Sharing Statement [file jamanetwopen-e2311308-s002.pdf]

Data Sharing Statement

Popkin. Fighting and Penalty Minutes Associated With Long-term Mortality Among National Hockey League Players, 1967 to 2022. *JAMA Netw Open*. Published May 10, 2023. doi:10.1001/jamanetworkopen.2023.11308

Data
Data available: Yes
Data types: Deidentified participant data, Data dictionary
How to access data: crm224@cumc.columbia.edu for deidentified data and data dictionary
When available: With publication

Supporting Documents
Document types: None

Additional Information
Who can access the data: researchers whose proposed use of the data has been approved
Types of analyses: continued research into the impact of fighting on mortality in professional ice hockey players
Mechanisms of data availability: with investigator support after approval of a proposal
